# High co-expression of TNF-α and CARDS toxin is a good predictor for refractory *Mycoplasma pneumoniae* pneumonia

**DOI:** 10.1186/s10020-019-0105-2

**Published:** 2019-08-09

**Authors:** Gang Li, Liping Fan, Yuqing Wang, Li Huang, Meijuan Wang, Canhong Zhu, Chuangli Hao, Wei Ji, Hansi Liang, Yongdong Yan, Zhengrong Chen

**Affiliations:** Department of Respiratory medicine, Children’s Hospital of Soochow University, Jingde Road NO.303, Suzhou, 215003 Jiangsu Province China

**Keywords:** TNF-α, CARDS toxin, M. pneumoniae pneumonia, RMPP

## Abstract

**Background:**

Early distinction between refractory *M. pneumoniae* pneumonia (RMPP) and non-RMPP (NRMPP) is still difficult. The community-acquired respiratory distress syndrome (CARDS) toxin can induce inflammatory and histopathological phenotypes associated with *M. pneumoniae* infection. This study aimed to investigate the clinical significance of CARDS toxin and pro-inflammatory cytokines in children with RMPP and to explore whether CARDS toxin can induce TNF-α expression.

**Methods:**

Levels of CARDS toxin and cytokines in BALF from control and children with MPP were determined by real-time PCR and ELISA, respectively. A receiver-operating characteristic (ROC) analysis was performed to assess the diagnostic values of CARDS toxin, TNF-α, and IL-6 in RMPP. The recombinant CARDS toxin was constructed and prepared at different concentrations for stimulation of RAW264.7 cells. After co-culture with CARDS toxin, cytokines were detected by ELISA and the mRNA levels were measured by real-time PCR. Effects of CARDS toxin and TNF-α on inflammatory cell infiltration and mucus secretion in mouse lungs were also evaluated.

**Results:**

Levels of CARDS toxin, TNF-α and IL-6 in bronchoalveolar lavage fluid (BALF) were significantly higher in RMPP cases compared with NRMPP cases. Furthermore, TNF-α had better diagnostic ability for differentiation of RMPP with AUC of 0.824 and Youden index of 0.692 compared with CARDS toxin and IL-6. Moreover, CARDS toxin was positively correlated with TNF-α level in MPP cases. In vitro assay revealed that CARDS toxin induced RAW264.7 macrophages to secrete TNF-α. Further in vivo assay showed that TNF-α deletion partially abrogated the CARDS toxin-mediated induction of inflammatory cell infiltration and mucus secretion in mouse lungs.

**Conclusions:**

The high co-expression of TNF-α and CARDS toxin in BALF is a good diagnostic biomarker for differentiating children with RMPP and NRMPP.

## Background

*Mycoplasma pneumoniae* pneumonia (MPP) caused by *Mycoplasma pneumoniae* (*M. pneumoniae*) infection is one of the most common forms of community-acquired pneumonia (CAP) in children (Wang et al., 2005; Gao et al., 2018). Although *M. pneumoniae* infection is usually considered as a self-limited process, sometimes it may cause various severe complications such as arthritis and necrotizing pneumonia (Zhang et al., 2016; Barreira et al., 2009; Azumagawa et al., 2008). Macrolides are the first-choice antibiotics for *M. pneumoniae* infections for children (Zhang et al., 2016). However, there are still some cases showing clinical and radiological deterioration in spite of macrolide antibiotic therapy for 7 days or longer, which was defined as refractory *M. pneumoniae* pneumonia (RMPP) (Zhang et al., 2016). The causes of RMPP are multiple, which include macrolide resistance, infections with other pathogen and immune disorder (Zhang et al., 2016; Barreira et al., 2009; Azumagawa et al., 2008; Ding et al., 2018). RMPP cases present with more frequent occurrences of cough, fever, and abnormal lung signs compared with non-RMPP (NRMPP) cases, and can develop into a severe life-threatening pneumonia (Ding et al., 2018). Therefore, it is important for pediatricians to recognize RMPP earlier and treat it promptly and prevent the progress of the disease.

*M. pneumoniae*, a leading pathogen causing CAP in children, shows cytotoxicity through expressing some pathogenic factors including community-acquired respiratory distress syndrome (CARDS) toxin (MPN372) (Becker et al., 2015). CARDS toxin is a 591-amino-acid ADP-ribosylating and vacuolating protein (Shimizu, 2016). CARDS toxin can induce and mimic major inflammatory and histopathological phenotypes associated with *M. pneumoniae* infection in rodents and primates (Ramasamy et al., 2018). However, CARDS toxin has currently only been studied in animal experiments, and there are few clinical studies concerning the correlation between CARDS toxin and children with MPP, especially RMPP.

A previous study has demonstrated that both mice and baboon responded to exposure to recombinant CARDS toxin by increasing the expression of the pro-inflammatory cytokines such as Interleukin (IL)-6 and tumor necrosis factor (TNF)-α (Hardy et al., 2009). TNF-α is mainly produced by monocytes and macrophages, and participates in the lung injury associated with severe pneumonia (Ding et al., 2018). Recent studies have shown several new biomarkers used for etiologic diagnosis in children with CAP (Principi & Esposito, 2017; Sungurlu & Balk, 2018; Liu et al., 2018). Some of these biomarkers have also been used for evaluating the *M. pneumoniae* infection severity (Chkhaidze & Kapanadze, 2017). Serum level of TNF-α has shown to be significantly higher in children with MPP compared with healthy children (Wang et al., 2005). However, whether TNF-α can be used as a biomarker for RMPP has not been defined.

Therefore, this study aimed to investigate the clinical significance of CARDS toxin and pro-inflammatory cytokines including TNF-α in bronchoalveolar lavage fluid (BALF) and explored predicting factors of RMPP in children. In addition, we explored whether the potential mechanism of TNF-α upregulation was associated with CARDS toxin.

## Methods

### Patients and study design

From January 2015 to June 2016, cases with MPP confirmed byboth positive enzyme-linked immunosorbent assay (ELISA) and polymerase chain reaction (PCR) were enrolled (*N* = 71). All cases were from 1 month to 14 years old and had fever, cough, tachypnea, chest retractions, abnormal auscultatory findings and radiologic evidence of CAP. Cases were not included if they had chronic lung disease, immunodeficiency, bronchopulmonary malformation, or co-infection.

Among these 71 children with MPP, 21 were diagnosed with RMPP and 50 with NRMPP. The RMPP was defined as showing a prolonged high degree fever (> 38.5 °C), radiological deterioration (increased infiltration area compared to first radiological finding) after macrolide combined oral prednisolone therapy (Azithromycin, 10 mg/d and Prednisolone, 1–2 mg/d) for 7 days or more (Tamura et al., 2008). Other children were defined as NRMPP.

A total of 22 children with bronchial foreign bodies who underwent foreign body removal in our hospital at the same time were selected as the control group. The inclusion criteria of the control group were: They had (1) a clear history of foreign body inhalation and a history of an irritating cough; (2) no inflammatory changes suggested by chest radiographs; (3) no respiratory tract infection within 32 months; (4) no history of application of hormones and immunosuppressive agents.

The study was approved by the Ethics Committee of the Children’s Hospital of Soochow University. Informed consent was obtained from parents or guardians of the children.

### Serology of *M. pneumoniae*

The specific antibodies against *M. pneumoniae* (IgM and IgG) were detected in serum samples (2 mL) of children during the acute phase (upon admission) and convalescent phase (upon discharge) respectively using a commercial ELISA kit (Serion ELISA classic *M. pneumoniae* IgG/IgM, Institute Virion/Serion, Würzburg, Germany), according to the manufacturer’s instructions. Acute *M. pneumoniae* infection was defined with either a single positive serum IgM titer or a 4-fold increase in IgG titer in the convalescent serum sample (Ding et al., 2018).

### Real-time fluorescent quantitation PCR for *M. pneumoniae* gene detection

The procedure of PCR for *M. pneumoniae* 16S rRNA detection was performed as described previously (Wang et al., 2014). Briefly, after being shaken for 30 s, one of the equally divided samples of BALF was centrifuged at 15,000×*g* for 5 min. After that, the sediment was collected and extraction of DNA from a 400-μL sample was performed. The DNA was then amplified using specific primers and probe. The sequences of the primers and probe were as follows: *M. pneumoniae*, forward: 5′-GCAAGGGTTCGTTATTTG-3′; reverse: 5′-CGCCTGCGCTTGCTTTAC-3′ (amplicon size: 380 bp); *M. pneumoniae*-probe: 5′-AGGTAATGGCTAGAGTTTGACTG-3′. The real-time PCR was performed using the iQ5TM BIO-icycler (Bio-Rad, Hercules, CA, USA). The PCR conditions were as follows: 37 °C for 2 min; initial denaturation at 94 °C for 10 min, followed by 40 cycles of denaturation at 94 °C for 10 s, annealing at 55 °C for 30 s, and extension at 72 °C for 40 s. Quantification curves were plotted using standard control samples at several concentrations (Daan gene Co. Ltd., Guangzhou, China). For each assay, a negative quality control, a critical quality control, a positive quality control, and four positive quantity controls (10^5^ copies/mL, 10^6^ copies/mL, 10^7^ copies/mL, and 10^8^ copies/mL) were used.

### Collection of clinical data

The age, sex, the duration of hospital stay and fever, the occurrence of pleural effusion, mucus plug and complications, the laboratory test data, and the effectiveness of treatment with macrolide were collected upon hospital admission and at patient discharge (Tables 1 and 2). All MPP children enrolled presented with or without mucus plug and pleural effusion on chest radiography.

**Table 1:** Demographic data and clinical characteristics in children with RMPP and NRMPP

Clinical parameters	MPP cases	Controls	*P* value	RMPP	NRMPP	*P* value
	(n = 71)	(*n* = 22)		(*n* = 21)	(*n* = 50)	
Age,months	64.6 ± 32.0	62.0 ± 29.2	0.733	64.5 ± 27.6	64.6 ± 34.0	0.986
Male, n(%)	29 (40.8)	11 (50)	0.449	11 (52.4)	18(36)	0.2
Hospital stay, days	10.1 ± 4.1	N/A		12.6 ± 5.5	9.0 ± 2.8	< 0.001
Duration of fever, days	8.2 ± 4.4	N/A		12.5 ± 3.1	6.4 ± 3.4	< 0.001
pleural effusion, n(%)	29 (40.8)	N/A		16 (76.2)	13 (26)	< 0.001
mucus plug, n(%)	21 (29.6)	N/A		15 (71.4)	6 (12)	< 0.001
expulmonary complications, n(%)	13 (18.3)	N/A		7 (33.3)	6 (12)	0.047
WBC, 10^6^/L	9.2 ± 3.7	N/A		9.0 ± 1.7	9.2 ± 4.2	0.766
CRP, mg/L	14.9 (4.7–34.9)	N/A		23.3 (10.3–44.6)	12.8 (4.4–31.3)	0.03
LDH, IU/L	480.9 ± 162.5	N/A		581.5 ± 179.7	437.2 ± 134.4	0.001
Lymphocytes in blood		N/A				
CD3+, %	67.0 ± 9.4	N/A		68 ± 8.2	66.6 ± 10.0	0.595
CD3 + CD4+, %	36.1 ± 9.3	N/A		35.3 ± 7.6	36.5 ± 10.0	0.654
CD3 + CD8+, %	26.4 ± 7.2	N/A		27.3 ± 6.0	26.0 ± 7.6	0.494
CD3-CD(16 + 56+), %	10.9 ± 7.8	N/A		9.7 ± 7.7	11.4 ± 7.8	0.481
CD3-CD19+, %	19.9 ± 8.5	N/A		20.1 ± 7.2	19.9 ± 9.1	0.904

*RMP* refractory *Mycoplasma pneumoniae* pneumonia, *NRMPP* non-refractory *Mycoplasma pneumoniae* pneumonia, *WBC* white blood cells, *CRP* C-reative protein, *LDH* lactate dehydrogenase, *CD* cluster of differentiation

**Table 2:** Comparison of CARDS toxin and cytokines in BALF from control and MPP (including RMPP and NRMPP) children

CARDS toxin and cytokines in BALF	MPP cases (*n* = 71)	Controls (*n* = 22)	*P* value	RMPP (*n* = 21)	NRMPP (*n* = 50)	*P* value
CARDS toxin, relative expression	12.5 (4.9–31.1)	0.5 (0.2–1.4)	< 0.001	19.6 (9.0–63.0)	10.0 (3.1–25.5)	0.025
TNF-α, pg/ml	68 ± 12.7	40.4 ± 6.2	< 0.001	76.9 ± 11.2	64.2 ± 11.4	< 0.001
IFN-γ, pg/ml	541.2 ± 104.1	357.3 ± 83.3	< 0.001	535.6 ± 96.9	543.5 ± 104.8	0.772
IL-36, pg/ml	429.9 ± 60.6	360.5 ± 61.2	< 0.001	438.8 ± 61.6	426.5 ± 60.5	0.457
IL-17, pg/ml	23.6 ± 5.6	24.7 ± 5.4	0.428	23.6 ± 5.4	23.6 ± 5.8	0.986
IL-8, pg/ml	67 ± 15.0	52.2 ± 14.7	< 0.001	65.4 ± 14.7	67.7 ± 15.2	0.584
IL-6, pg/ml	24.7 ± 5.7	16.1 ± 4.0	< 0.001	28.1 ± 3.8	23.2 ± 5.8	< 0.001
IL-4, pg/ml	66.8 ± 14.6	36.4 ± 6.5	< 0.001	68.7 ± 12.5	66.0 ± 15.4	0.477

*RMP* refractory *Mycoplasma pneumoniae* pneumonia, *NRMPP* non-refractory *Mycoplasma pneumoniae* pneumonia, *CARDS toxin* community-acquired respiratory distress syndrometoxin, *BALF* bronchoalveolar lavage fluid, *TNF-α* tumor necrosis factor-α, *IFN-γ* interferon-γ, *IL-36* interleukin-36, *IL-17* interleukin-17, *IL-8* interleukin-8, *IL-6* interleukin-6, *IL-4* interleukin-4

Peripheral venous blood (2–3 mL) was collected within 24 h from children in each group. The blood sample was immediately sent for the following laboratory examination: the complete blood count, C-reactive protein (CRP), lactate dehydrogenase (LDH), subpopulations of T lymphocytes, andspecific antibody to *M. pneumoniae* and other tests (Table 1).

Flexible fiber optic bronchoscopy and bronchoalveolar lavage were performed following the guidelines described previously (Wang et al., 2017a). The BALF was gently aspirated, collected and prepared for detection of *M. pneumoniae* DNA, CARDS toxin, and cytokines. The cytokines including TNF-α, interferon-γ (IFN-γ), IL-36, IL-17, IL-8, IL-6, and IL-4 in the supernatants of BALF were determined using their commercial ELISA kits (R&D Systems, Minneapolis, MN, USA) according to the manufacturer’s instructions (Table 2). All children were treated with alveolar irrigation and drainage using a fiberoptic bronchoscope as previously described (Xu et al., 2011).

### Real-time PCR

The relative expression of CARDS toxin in BALF from children was determined by real-time PCR. Briefly, total RNA was extracted from BALF using TRIzol reagent (Invitrogen, Waltham, MA, USA) according to the manufacturer’s protocol. RNA was then reverse transcribed into cDNAs using RNA Transcription Kit (Promega, USA). The cDNA templates were amplified by qRT-PCR using SYBR Green PCR Mix (TaKaRa, Japan). The primer sequences were as follows: CARDS toxin: forward, 5′-TTCCACTTCAGAAACACCCACAGC-3′, reverse, 5′- TCAATCAGGGCACGCAAACG-3′; pdhA (internal control for CARDS toxin): forward, 5′-ACTGGTTCTGCCCTACCTTCCGTTCC-3′, reverse, 5′-CTTCGTGCATTGCTTCGTAACTCGC-3′. The relative expression of CARDS toxin was calculated by the 2^-ΔCt^ method and normalized to the internal control pdhA.

The mRNA level of TNF-α in RAW264.7 cells was determined by real-time PCR as previously described (Ma et al., 2016). The primer sequences were as follows: TNF-α: forward, 5′- AGCCGATGGGTTGTACCT-3′, reverse, 5′- TGAGTTGGTCCCCCTTCT-3′; 18S: forward, 5′- GCCCGAGCCGCCTGGATAC-3′, reverse, 5′- CCGGCGGGTCATGGGAATAAC-3′. TNF-α mRNA level was calculated by the 2^-ΔΔCt^ method and normalized to the internal control 18S.

### Construction of recombinant CARDS toxin

The full-length gene sequence and protein sequence of MPN372 were obtained from the NCBI database. *M. pneumoniae* uses TGA to encode tryptophan and TGA is a termination codon in most other species. Therefore, we mutated eight codon TGAs encoding tryptophan into TGG. The optimized MPN372 gene sequence was cloned into the pFastBac donor plasmid vector for virus packaging. High-Five cells in logarithmic growth phase were infected with high titer recombinant virus to express the target protein, which was further purified by nickel column.

### Vacuolization induction by CARDS toxin

HeLa cells were purchased from ATCC (Manassas, VA, USA) and cultured in RPMI-1640 medium supplemented with 10% fetal calf serum (FCS) in a 37 °C humidified incubator with air and 5% CO_2_. When the cell confluence reached 60–70%, the culture medium was replaced with fresh RPMI-1640 medium containing 50 μg/mL CARDS toxin. The medium without CARDS toxin served as the negative control. After 24 h and 48 h of incubation, the ability of CARDS toxin to induce vacuolization in HeLa cells was evaluated under a microscope (Olympus BH-2; Olympus Corporation, Tokyo, Japan).

### RAW264.7 cell stimulation by CARDS toxin in vitro

The murine macrophage cell line RAW264.7 was purchased from ATCC. RAW264.7 cells were cultured in RPMI-1640 medium, supplemented with 10% heat-inactivated FCS, 2 mM L-glutamine, 100 U/mL penicillin, and 100 μg/mL streptomycin, at 37 °C under 5% CO_2_. The cell density was adjusted to 2 × 10^5^ cells/ml in 12-well plates. When the cell confluence reached about 50%, the culture medium was replaced with fresh RPMI-1640 medium containing different concentrations of CARDS toxin (0, 5, and 50 μg/mL). After 48 h of incubation, the cells were harvested and the levels of 13 cytokines including TNF-α, IFN-γ, IL-2, IL-4, IL-5, IL-6, IL-9, IL-10, IL-13, IL-17A, IL-17F, IL-21, and IL-22 in the supernatant were measured using LEGENDplex™ Mouse Th Cytokine Panel (13-plex) (BioLegend, San Diego, CA, USA) according to the manufacturer’s instructions.

### Animals and in vivo experiments

The animal used in this experiment were approved by the Ethics Committee of Children’s Hospital of Soochow University. BALB/c mice (age of 6–7 weeks, weighing 20 ± 2 g) were purchased from the Laboratory Animal Center of Soochow University, Suzhou, China. TNF-α^−/−^ mice (BALB/c background) were purchased from Jackson Laboratory (Bar Harbor, ME, USA). All animals were kept in separate cages and housed under constant temperature (25 ± 1 °C) and humidity (50%) with 12 h light-dark cycles, and had free access to food and water.

CARDS toxin (50 μg/0.5 mL) or equivalent PBS was intratracheally instilled into wild-type BALB/c mice and TNF-α^−/−^ mice (*N* = 5/group). On the 7th day, the mice were sacrificed by cervical dislocation under anesthesia and lung tissues were harvested for histological examination.

### Histological examination

Heamatoxylin and eosin (H&E) staining was performed to observe the infiltration of inflammatory cells in the lungs. Periodic acid-Schiff (PAS) staining was performed to observe the secretion of bronchial mucus. Briefly, mouse lung tissues were fixed with 4% formaldehyde, embedded in paraffin and cut to obtain serial 4-μm thick sections. Subsequently, these sections were stained with H&E (Yeasen, Shanghai, China) and PAS (Solarbio, Beijing, China) following routine staining procedures. The pathologic changes in these tissues were observed under a light microscope (Olympus BH-2; Olympus Corporation, Tokyo, Japan). H&E average score and the percentage of PAS-positive (PAS^+^) epithelial cells were analyzed by a pathologist in a blinded fashion.

### Statistical analysis

All analyses were performed using SPSS for Windows (version 21.0; SPSS Inc., Chicago, IL, USA). Categorical data were analyzed using the Chi-square test. Continuous variables were analyzed using Student’s*t*-test or the Mann-Whitney U-test if the data had a non-normal distribution. For in vitro studies, statistical significance was determined using one-way ANOVA. Correlations between CARDS toxin and TNF-α or IL-6 were evaluated by Spearman correlation test. Data were expressed as means ± standard deviation (SD) if data had a normal distribution. *p* < 0.05 was considered statistically significant.

## Results

### Demographic data and clinical characteristics in children with RMPP and NRMPP

As shown in Table 1, there was no significant difference in mean age and gender between control and MPP children. Of note, the RMPP cases had longer hospital stays and duration of fever than the NRMPP cases (all *p* < 0.05). The occurrences of pleural effusion, mucus plug, and expulmonary complications were more frequent in RMPP cases when compared with NRMPP cases (all *p* < 0.05). Furthermore, levels of CRP and LDH in peripheral blood were significantly higher in RMPP cases compared with NRMPP cases (*p* < 0.05). However, there was no significant difference in WBC and lymphocytes distributions between RMPP and NRMPP cases.

### Comparison of CARDS toxin and cytokines in BALF from control and MPP children

As shown in Table 2, the relative expression of CARDS toxin and levels of TNF-α, IFN-γ, IL-36, IL-8, IL-6, and IL-4 in BALF from MPP cases were significantly higher when compared with control cases (all *p* < 0.05). However, there was no significant difference in IL-17 levels in BALF between the control and MPP cases. Furthermore, the relative expression of CARDS toxin and levels of TNF-α and IL-6 in BALF were significantly higher in RMPP cases compared with NRMPP cases (all *p* < 0.05). However, there was no significant difference in levels of IFN-γ, IL-36, IL-8, IL-4, and IL-17 in BALF between RMPP and NRMPP cases.

### Comparison of CARDS toxin expression between MPP cases with/without plug and pleural effusion

**Fig. 1:**
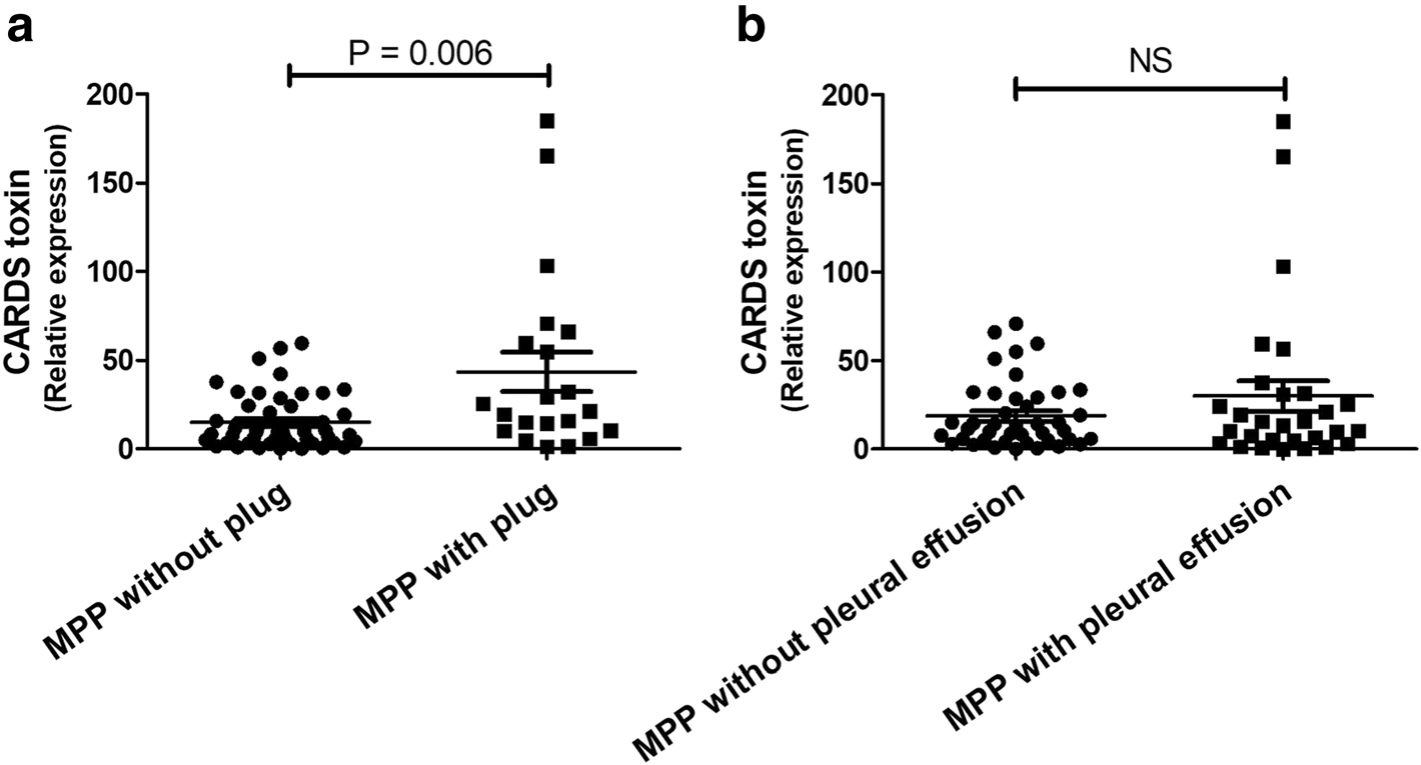
Comparison of CARDS toxin expression between MPP cases with/without plug (**a**) and pleural effusion (**b**). Children with MPP were divided into MPP with plug group (*N* = 50) and MPP without plug group (*N* = 21), according to the presence or absence of mucus plugs. They were also divided into MPP with pleural effusion group (*N* = 42) and MPP without pleural effusion (*N* = 29) group, according to results of imageological examination. The expression of CARDS toxin in bronchoalveolar lavage fluid (BALF) in each group was measured. NS, no significance

Data revealed that the expression of CARDS toxin was significantly higher in BALF from children with MPP with mucus plug than that in children with MPP without plug (*p* = 0.006; Fig. 1a). However, no significant difference in CARDS toxin was observed in BALF from children between MPP with pleural effusion and MPP without pleural effusion (Fig. 1b).

### CARDS toxin was positively correlated with TNF-α level in MPP cases

**Fig. 2:**
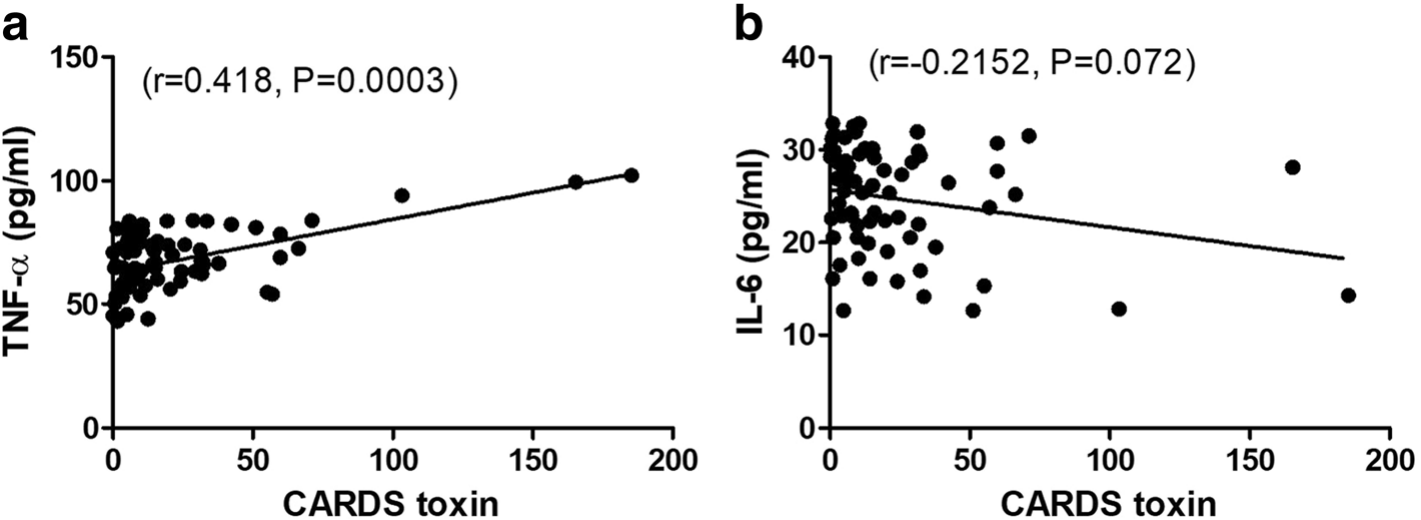
CARDS toxin was positively correlated with TNF-α level in BALF. The positive correlation between CARDS toxin and TNF-α (**a**), and a negative correlation between CARDS toxin and IL-6 in BALF from children with MPP (**b**)

We next analyzed the correlations between CARDS toxin and pro-inflammatory cytokines TNF-α and IL-6 in BALF from children with MPP. Data showed that TNF-α level was positively correlated with CARDS toxin level (r = 0.418, *p* = 0.0003; Fig. 2a). In contrast, IL-6 level was negatively correlated with CARDS toxin level (r = − 0.2152, *p* = 0.072; Fig. 2b).

### Diagnostic values of CARDS toxin, TNF-α, and IL-6 in children with RMPP

**Fig. 3:**
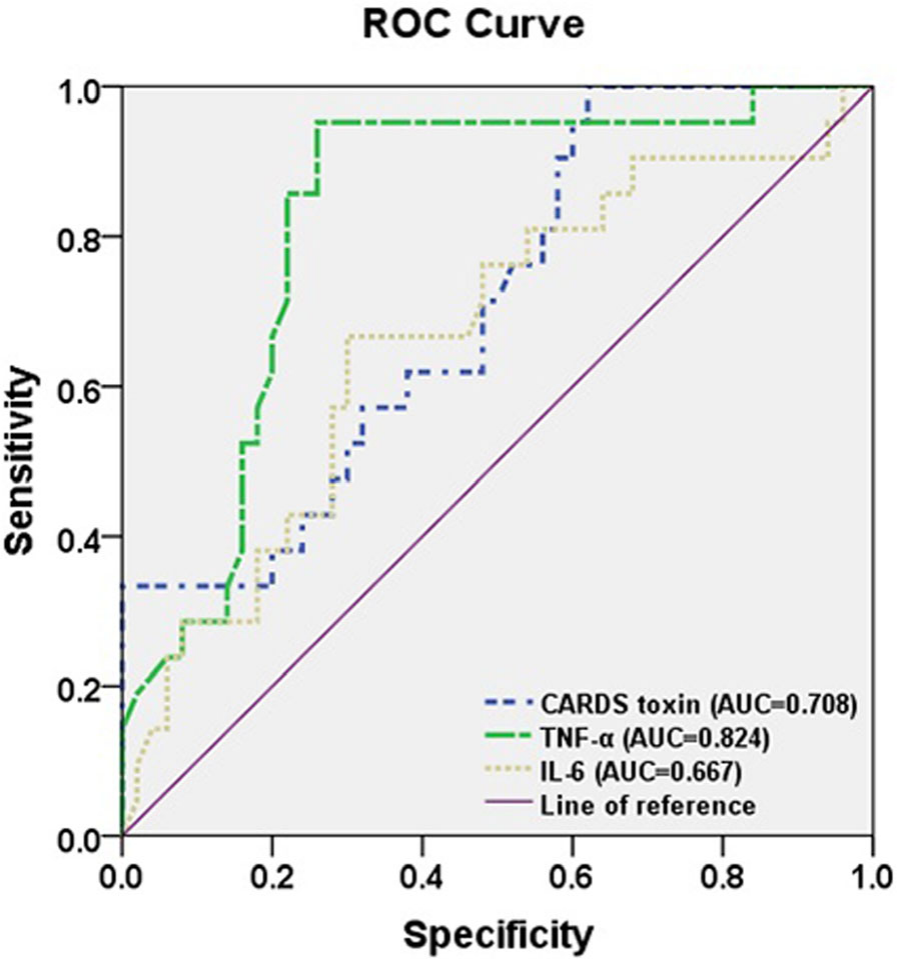
Diagnostic values of CARDS toxin, TNF-α, and IL-6 in children with RMPP. TNF-α had better diagnostic ability for differentiation of RMPP with the best cut-off of 68.25 pg/ml, AUC of 0.824 and Youden index of 0.692 compared with CARDS toxin and IL-6

To estimate the diagnostic abilities of CARDS toxin, TNF-α, and IL-6 in children with RMPP, a receiver-operating characteristic (ROC) analysis was performed. As shown in Fig. 3, TNF-α had better diagnostic ability for differentiation of RMPP with the best cut-off of 68.25 pg/ml, AUC of 0.824 and Youden index of 0.692 compared with CARDS toxin and IL-6.

### Plasmid construction and vacuolation activity verification of CARDS toxin

**Fig. 4:**
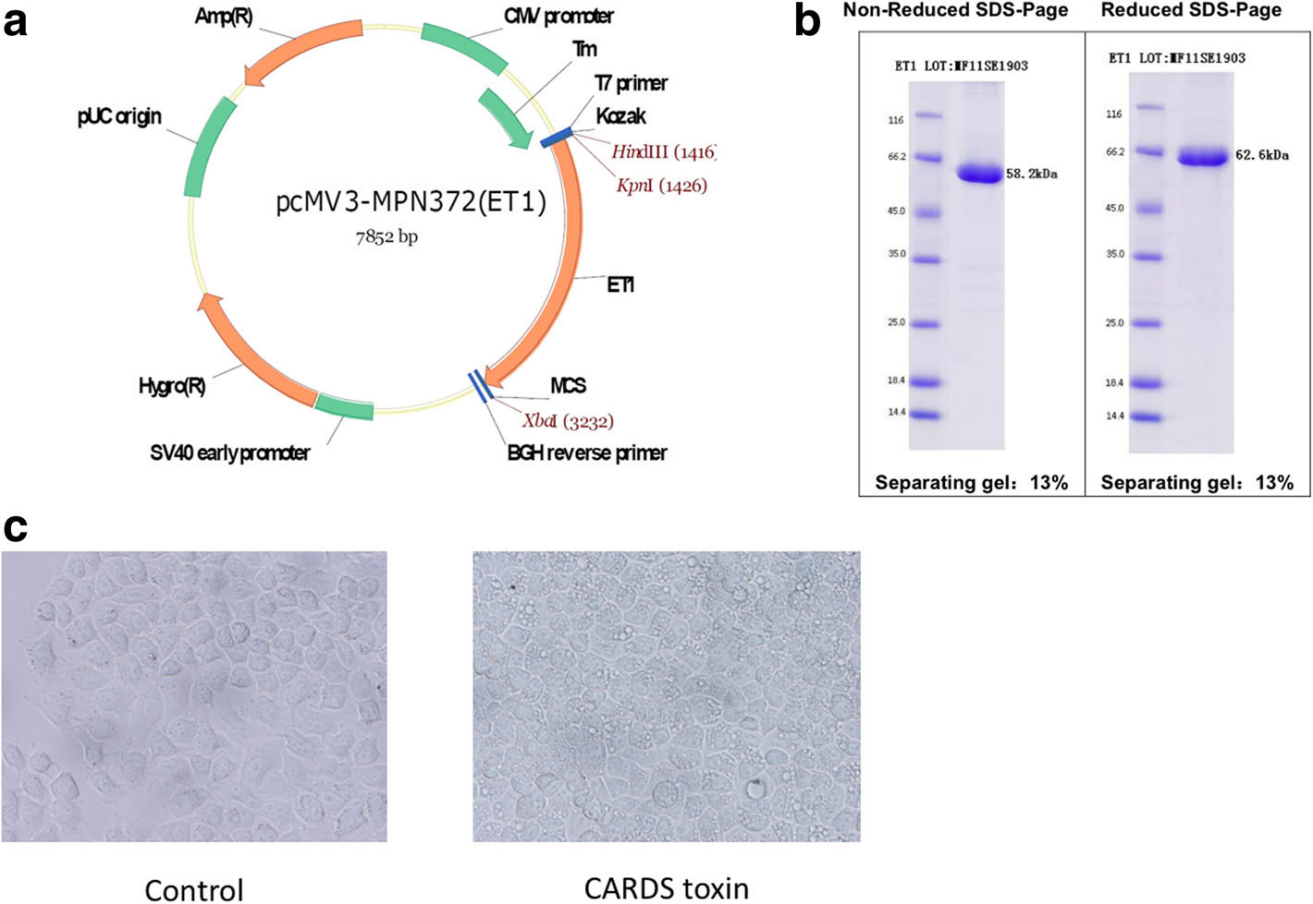
Plasmid construction and vacuolation activity verification of CARDS toxin. **a** Schematic diagram of plasmid construction. CARDS toxin gene (MPN372) eukaryotic expression vector was constructed and purified. **b** Purity of CARDS toxin by SDS-PAGE. **c** HeLa cells were co-cultured with 50 μg/mL CARDS toxin or not for 24 h. CARDS toxin exhibited the ability to induce vacuolization in HeLa cells compared with the control group.

The recombinant CARDS toxin was constructed (Fig. 4a) and the purity of CARDS toxin was checked by SDS-PAGE (Fig. 4b). The bioactivity of CARDS toxin was determined by the toxin’s ability to induce vacuolization in HeLa cells. Compared with the control group, CARDS toxin exhibited the ability to induce vacuolization in HeLa cells (Fig. 4c). These results confirmedthe bioactivity of CARDS toxin based on the ability to produce vacuoles in epithelial cells.

### CARDS toxin induced RAW264.7 macrophages to secrete TNF-α

**Fig. 5:**
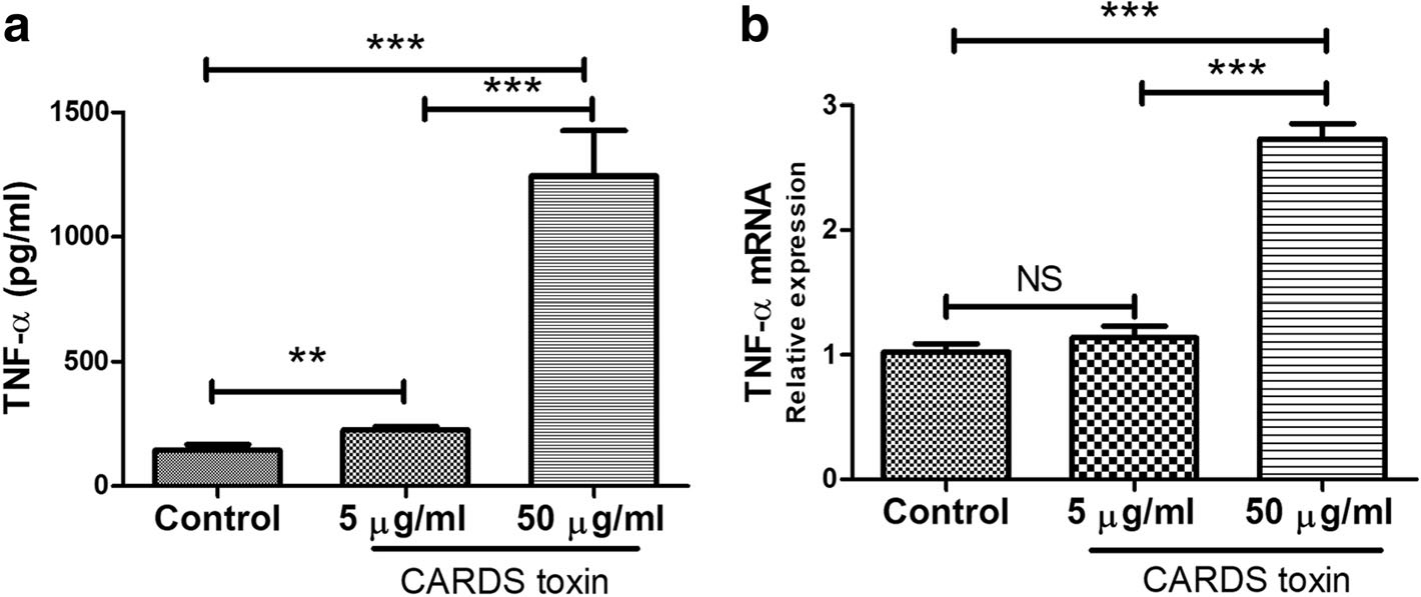
CARDS toxin induced RAW264.7 macrophages to secrete TNF-α. RAW264.7 macrophages were co-cultured with different concentrations of CARDS toxin (0, 5, and 50 μg/mL) for 48 h. **a** TNF-α level secreted from RAW264.7 cells was determined by ELISA. **b** TNF-α mRNA level in RAW264.7 cells was determined by real-time PCR. NS, no significance; ^**^p < 0.01, ^***^p < 0.001

The levels of 13 cytokines including TNF-α, IFN-γ, IL-2, IL-4, IL-5, IL-6, IL-9, IL-10, IL-13, IL-17A, IL-17F, IL-21, and IL-22 in the supernatant of CARDS toxin-stimulated RAW264.7 cells were measured using ELISA. Data revealed that CARDS toxin significantly increased TNF-α level secreted from RAW264.7 macrophages in a dose-dependent manner (*p* < 0.05), whereas it had no significant effect on levels of the other cytokines (Fig. 5a). The data of real-time PCR further confirmed that TNF-α mRNA level in RAW264.7 cells was notably upregulated by 50 μg/mL CARDS toxin (*p* < 0.05; Fig. 5b).

### Effect of CARDS toxin on inflammatory cell infiltration and mucus secretion in mouse lungs

**Fig. 6:**
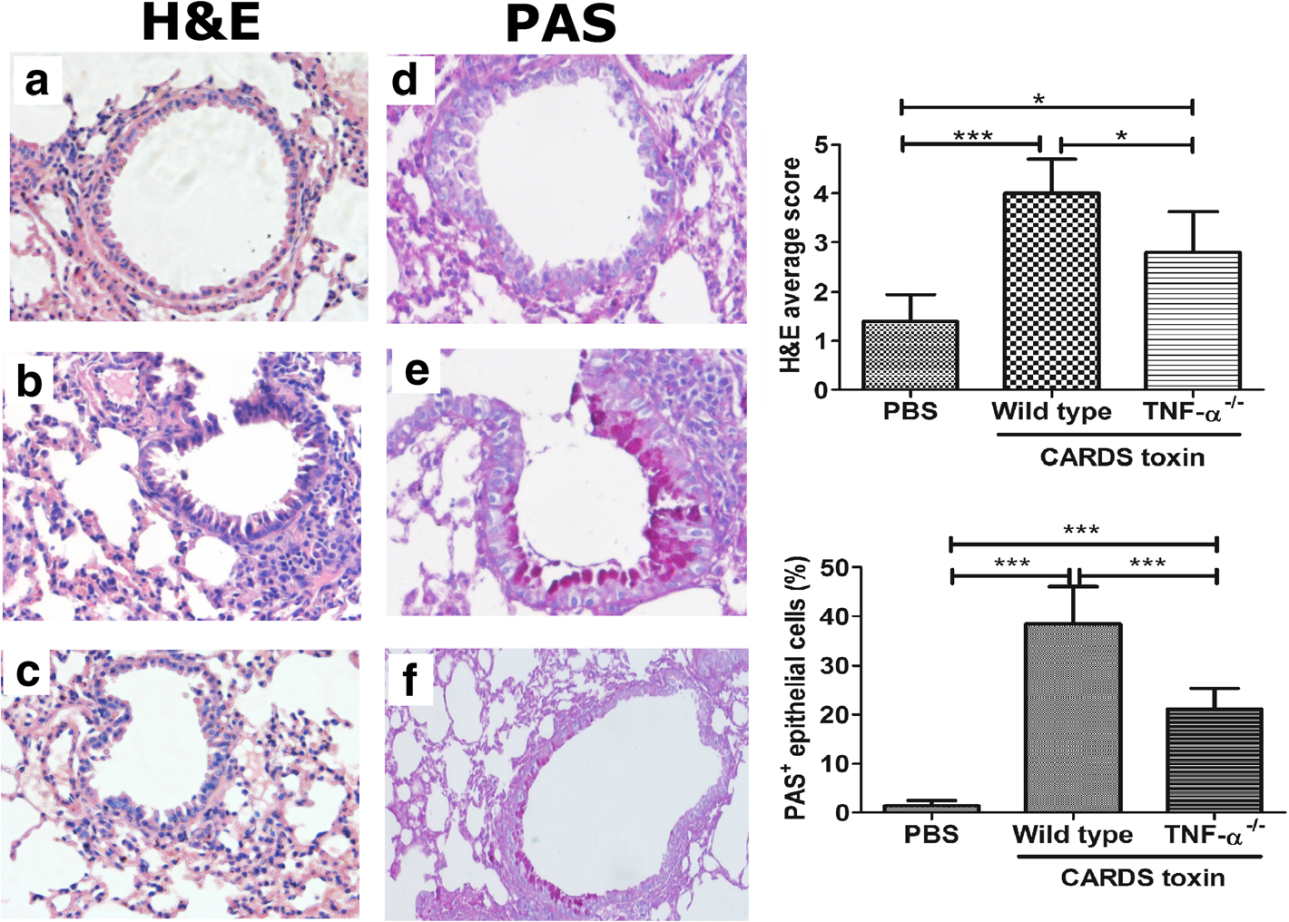
Effect of CARDS toxin on inflammatory cell infiltration and mucus secretion in mouse lungs. Following intranasal instillation of CARDS toxin (50 μg/0.5 mL) or equivalent PBS, the mouse lung tissues were harvested for heamatoxylin and eosin (H&E) staining and Periodic acid-Schiff (PAS) staining on the 7th day. Ten bronchial tubes with an inner diameter of 150–200 μm were randomly selected from each section under a light microscope. H&E average score and the percentage of PAS-positive (PAS^+^) epithelial cells in the total number of epithelial cells were analyzed by a pathologist in a blinded fashion. N = 5/group. ^*^p < 0.05, ^***^p < 0.001. **a** H&E staining in PBS controls; **b** H&E staining in CARDS toxin groups; **c** H&E staining in TNF-α^-/-^ mice stimulated with CARDS toxin; **d** PAS staining in PBS controls; **e** PAS staining in CARDS toxin groups; **f** PAS staining in TNF-α-/- mice stimulated with CARDS toxin

As shown in H&E staining, there was no obvious inflammatory cell infiltration around the airway and vascular tissue (Fig. 6a). Compared with the PBS group, the wild type mice treated with CARDS toxin showed airway tube cavity stenosis and a large amount of inflammatory cell infiltration around the airway and vascular tissue (Fig. 6b). Compared with wild type mice, the TNF-α^−/−^ mice treated with CARDS toxin showed a notable decrease in inflammatory cell infiltration (Fig. 6c).

As indicated in PAS staining, there were few PAS-positive epithelial cells in the airway in the PBS group (Fig. 6d). Compared with the PBS group, the wild type mice treated with CARDS toxin showed a significant increase in PAS-positive epithelial cells (i.e. goblet cell hyperplasia) and hyperactive mucus secretion (Fig. 6e). Compared with wild type mice, the TNF-α^−/−^ mice treated with CARDS toxin showed a notable decrease in PAS-positive epithelial cells (Fig. 6f). Taken together, these data indicated that TNF-α is involved in the CARDS toxin-mediated induction of inflammatory cell infiltration and mucus secretion in mouse lungs.

## Discussion

Although most of the children with *M. pneumoniae* infection could make remarkable recovery, the prevalence of RMPP has rapidly increased in recent years due to the abuse of macrolides and the emergence of antibiotic-resistant strains (Wang et al., 2017b). Thus, it is important for pediatricians to recognize RMPP earlier and treat it promptly. In this study, children with RMPP had longer hospital stays and duration of fever, more frequent occurrences of pleural effusion, mucus plug, and expulmonary complications, as well as significantly higher levels of CRP and LDH in peripheral blood, compared with NRMPP cases.

Studies have provided the possibility to determine the extent of pneumonia damage by detecting the amount of CARDS toxin in the clinic.Introduction of recombinant CARDS toxin to the airways of mice caused changes in airway function, cytokine expression, and cellular inflammation, which are consistent with what has been reported for *M. pneumoniae* infection in animal models (Hardy et al., 2009). The concentration of recombinant CARDS toxin in BALF directly correlated with the number of mycoplasma genomes and the degree of histologic pulmonary inflammation and injury in mice (Kannan et al., 2011). However, there are few clinical studies concerning the correlation between CARDS toxin and children with MPP, especially RMPP.

CARDS toxin is a pathogenic virulence factor in *M. pneumoniae* infection (Segovia et al., 2018). In this study, a significant increase in CARDS toxin expression from BALF was observed in both MPP cases compared with control cases and in RMPP cases compared with NRMPP cases. Furthermore, CARDS toxin was significantly higher in BALF from children with MPP with mucus plug than that in children with MPP without plug. Our data reinfore the pathogenic role of CARDS toxin in MPP, especially in RMPP with mucus plug.

Immune disorders caused by *M. pneumoniae* infection are also one of the main factors of RMPP pathogenesis (Chkhaidze & Kapanadze, 2017; Wang et al., 2014). Introduction of CARDS toxin caused damage to mouse lung tissues, accompanied by the increase of the expression of the pro-inflammatory cytokines (such as TNF-α and IL-6) and immune disorders (Hardy et al., 2009). These data indicated the potential association between CARDS toxin and cytokines and their pathogenic roles in MPP. Therefore, in this study, we explored the correlation between CARDS toxin and cytokines in BALF to further understand the immune status after *M. pneumoniae* infection. Here, we observed significantly higher levels of CARDS toxin, TNF-α, and IL-6 in BALF from both MPP cases compared with control cases and in RMPP cases compared with NRMPP cases. Furthermore, CARDS toxin was positively correlated with TNF-α level and negatively correlated with IL-6 level in BALF from MPP cases. Importantly, we evaluated the diagnostic values of CARDS toxin, TNF-α, and IL-6 in RMPP. Our ROC analysis showed that TNF-α had better diagnostic ability for differentiation of RMPP with the best cut-off of 68.25 pg/ml, AUC of 0.824 and Youden index of 0.692 compared with CARDS toxin and IL-6. Altogether, these data indicated that high expression of TNF-α may serve as a good predictor for RMPP.

In line with this, a recent study showed that the serum TNF-α level in children with MPP was significantly higher than that in healthy children (Wang et al., 2005). Furthermore, Wang et al. *(Ding et al.,* 2018*;* *Wang et al.,* 2014*)* observed that serum TNF-α levels in children with RMPP were significantly higher than those in children with NRMPP, which was confirmed in a mouse model of MPP (Hsia et al., 2012).

Our findings showing the positive correlation between CARDS toxin and TNF-α expression in BALF from all 71 MPP cases suggested that high expression of TNF-α in BALF may be induced by a high amount of CARDS toxin. To address this, we constructed recombinant CARDS toxin and verified its vacuolation activity in HeLa cells. Furthermore, our results revealed that recombinant CARDS toxin induced RAW264.7 macrophages to secrete TNF-α in a dose-dependent manner, whereas it had no significant effect on levels of the other cytokines. Finally, we verified in vivo, the role of CARDS toxin-TNF-α axis in lung inflammatory cell infiltration and mucus secretion. Our results demonstrated that TNF-α deletion partially abrogated the recombinant CARDS toxin-mediated induction of inflammatory cell infiltration and mucus secretion in mouse lungs. Additionally, the level of TNF-α in BALF has been shown to increase with increasing CARDS toxin exposure in mice (Hardy et al., 2009). Collectively, these findings indicated CARDS toxin induced TNF-α expression and thereby involved in enhancing lung inflammatory cell infiltration and mucus secretion.

## Conclusions

The TNF-α level is increased in BALF from children with RMPP and is a good diagnostic biomarker for differentiating RMPP and NRMPP. The high expression of TNF-α may be induced by CARDS toxin. This study provides a new basis for the early diagnosis and treatment of RMPP.

## Data Availability

The datasets generated during and/or analysed during the current study are available from the corresponding author on reasonable request.

## Abbreviations

BALF: Bronchoalveolar lavage fluid
CAP: Community-acquired pneumonia
CARDS: Community-acquired respiratory distress syndrome
CRP: C-reactive protein
ELISA: Enzyme-linked immunosorbent assay
IFN-γ: Interferon-γ
IL: Interleukin
LDH: Lactate dehydrogenase
*M. pneumoniae*: *Mycoplasma pneumoniae*
MPP: *Mycoplasma pneumoniae* pneumonia
NRMPP: Non-refractory *M. pneumoniae* pneumonia
PCR: Polymerase chain reaction
RMPP: Refractory *M. pneumoniae* pneumonia
TNF: Tumor necrosis factor
